# Effects of chronic alcohol exposure on ischemia–reperfusion-induced acute kidney injury in mice: the role of β-arrestin 2 and glycogen synthase kinase 3

**DOI:** 10.1038/emm.2017.76

**Published:** 2017-06-23

**Authors:** Lihua Wang, Yifei Zhu, Lili Wang, Jingjing Hou, Yongning Gao, Lei Shen, Jingyu Zhang

**Affiliations:** 1 Division of Blood Purification, The Second Hospital of Hebei Medical University, Shijiazhuang, China; 2 Department of Neurology, The Second Hospital of Hebei Medical University, Shijiazhuang, China; 3 Department of Hematology, The Second Hospital of Hebei Medical University, Key Laboratory of Hematology of Hebei Province, Shijiazhuang, Hebei, China

## Abstract

Little is known about the effects of chronic alcohol intake on the outcome of acute kidney injury (AKI). Hence, we examined the effects of chronic alcohol intake on the development of renal fibrosis following AKI in an animal model of bilateral renal ischemia–reperfusion (IR) injury. We first found that chronic alcohol exposure exacerbated bilateral IR-induced renal fibrosis and renal function impairment. This phenomenon was associated with increased bilateral IR-induced extracellular matrix deposition and an increased myofibroblast population as well as increased bilateral IR-induced expression of fibrosis-related genes in the kidneys. To explore the mechanisms underlying this phenomenon, we showed that chronic alcohol exposure enhanced β-arrestin 2 (*Arrb2*) expression and Akt and glycogen synthase kinase-3 (GSK3)β activation in the kidneys. Importantly, pharmacological GSK3 inhibition alleviated bilateral IR-induced renal fibrosis and renal function impairment. Furthermore, we demonstrated that *Arrb2*^−/−^ mice exhibited resistance to IR-induced renal fibrosis and renal function impairment following chronic alcohol exposure, and these effects were associated with attenuated GSK3β activation in the kidneys. Taken together, our results suggest that chronic alcohol exposure may potentiate AKI via β-arrestin 2/Akt/GSK3β-mediated signaling in the kidney.

## Introduction

Acute kidney injury (AKI) is a major clinical problem that can result in prolonged hospitalization, chronic renal failure and death.^1, 2, 3, 4, 5^ One of the leading causes of AKI is renal ischemia–reperfusion injury (IRI) due to surgical renal ischemia or renal hypoperfusion.^1, 2^ Importantly, evidence has shown that the severity and incidence of AKI have been increasing in recent years.^6^ Hence, studies are necessary to identify the cellular and molecular mechanisms underlying acute kidney injury in order to provide therapeutic targets for AKI prevention and therapy.

Although the associations between high alcohol consumption and the progression of kidney damage such as chronic kidney disease (CKD) remain controversial,^7^ it has been recognized that chronic alcohol intake can affect renal function. For example, alcoholic liver cirrhosis with associated hepato-renal syndrome is a common complication of chronic alcohol consumption and can result in renal failure because of extreme splanchnic vasodilatation and compensatory renal vasoconstriction.^8, 9^ In addition, evidence has suggested that a wide range of disturbances in electrolyte and acid–base balance can be observed in alcoholics because of alcohol-induced tubular dysfunction.^10, 11, 12, 13^ Therefore, we hypothesized that a history of chronic alcohol intake may worsen the consequences of AKI.

Renal IRI is a widely used animal model for the study of mechanisms underlying AKI. As a model of AKI, bilateral renal IRI can induce a significant increase in serum creatinine and blood urea nitrogen (BUN), which are also seen in patients with AKI.^6, 14^ While some studies have examined the long-term impact of bilateral renal IRI,^15, 16, 17^ most have indicated that kidney function and morphology can return to almost normal ~2 weeks after the initial mild bilateral renal IRI.^14, 18, 19^ As a result, the consequences of AKI induction are generally studied from 24 h to up to 2 weeks after the bilateral renal IRI.^19, 20, 21, 22^ Hence, we used a rodent bilateral renal IRI model in the present study to examine the effects of chronic alcohol intake on the consequences of mild AKI 2 weeks after injury.

One of the classic outcomes of AKI is renal fibrosis, which can develop as a consequence of maladaptive repair after acute kidney injury.^23, 24^ Importantly, previous studies have demonstrated that the glycogen synthase kinase-3 (GSK3) family of protein kinases plays a critical role in injury and repair of renal tubular epithelial cells after AKI. Specifically, GSK3β gene knockdown reduces AKI-induced apoptosis *in vitro* and *in vivo*.^25, 26^ Moreover, pharmacological inhibition of GSK3 reduces apoptosis and renal tubular injury in renal IRI.^25, 26, 27, 28, 29^ Furthermore, it has been shown that GSK3β is expressed in renal myofibroblasts,^30^ which are critical for the development of renal fibrosis following renal IRI. In fact, previous studies have shown that GSK3 inhibition can attenuate fibroblast activation and fibrosis development following renal IRI in mice.^30^ Given that previous studies have shown that chronic alcohol exposure results in GSK3β activation in the liver and brain,^31, 32, 33^ we hypothesized that GSK3 may be involved in the impact of chronic alcohol intake on the consequences of AKI.

While many studies have shown that β-arrestins play an important role in regulating G-protein-coupled receptor internalization,^34^ β-arrestins can also interact with many signaling molecules and serve as scaffold proteins.^35, 36^ Recent studies have shown that β-arrestin-2 can form a complex with Akt and protein phosphatase 2 (PP2A) to mediate the dephosphorylation of Akt, which in turn results in GSK3β activation.^37^ In addition, overexpression of β-arrestin-2 increases Akt-mediated activation of GSK3β in endometrial cancer cells.^38^ These studies suggested that Akt may mediate the effects of β-arrestin-2 on the activation of GSK3β in the kidney. To test these hypotheses, we first examined the effects of alcohol pre-conditioning on the development of renal fibrosis following AKI in an animal model of bilateral renal IRI. Furthermore, we examined the role of β-arrestin 2, Akt and GSK3β in the effect of chronic alcohol intake on the consequences of AKI.^39^

## Materials and methods

### Animals

**Figure 1:**
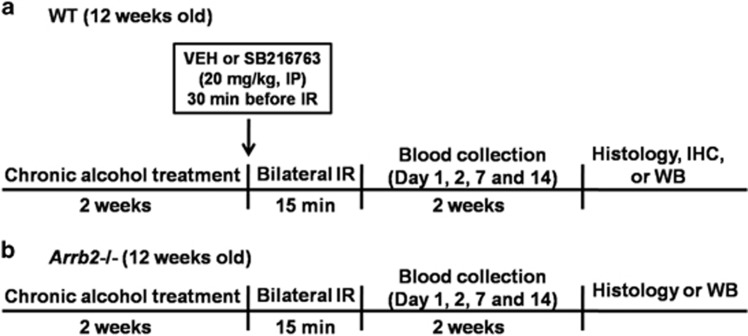
Experimental timeline. Twelve-week-old male β-arrestin-2 knockout mice and WT C57BL/6J control mice were used for the experiments. The mice were randomly separated into two treatment groups. (**a**) One group of mice received a dextrose-containing liquid diet, and the second group received a nutritionally complete liquid diet containing 4.5% (v/v) ethanol in their home cages for 2 weeks. Renal IRI was conducted by clamping both renal pedicles for 15 min under pentobarbital anesthesia (60 mg kg^−1^, i.p.), followed by reperfusion. (**b**) Sham groups of mice underwent surgery to expose the renal pedicles without clamping. Tail vein blood samples were collected to measure blood urea nitrogen (BUN) and creatinine levels. Histology, immunohistochemistry and western blot analyses were performed at 14 days post IRI. IRI, ischemia–reperfusion injury.

β-Arrestin-2 knockout (KO) mice generated in the laboratory of R.J. Lefkowitz (Duke University Medical Center, Durham, NC, USA) were back-crossed to C57BL/6J mice for 10 generations. Wild-type (WT) or β-arrestin KO littermates from heterozygous breeding and descendants resulting from homozygous breeding of WT or β-arrestin KO mice after the initial back-crossing were used. All mice were individually housed in plastic mouse cages with free access to standard rodent chow (Teklad, Madison, WI, USA) and water except when an ethanol diet was introduced. The colony room was maintained at ~22 °C with a 12-h light/dark cycle in which the lights were turned off at 0600 hours. In all experiments, 12-week-old male KO and WT control mice were used. The experimental timeline is shown in Figures 1a and b. All experiments were performed strictly in accordance with the National Institutes of Health Guide for the Care and Use of Laboratory Animals and were approved by the Animal Care and Use Committee of The Second Hospital of Hebei Medical University.

### Chronic alcohol administration

The mice were randomly separated into two treatment groups. One group of mice received a dextrose-containing liquid diet, and a second group received a nutritionally complete liquid diet containing 4.5% (v/v) ethanol (Abbott Laboratories, Abbott Park, IL, USA) in their home cages for 2 weeks. The diet used was a lactalbumin/dextrose-based, nutritionally complete diet with concentrations of vitamins, minerals and other nutrients derived from ICN Research Diets.^40, 41, 42^ The dextrose calories in the normal diet were equated with the ethanol calories in the alcohol diet. Water was provided in a second bottle *ad libitum* throughout the treatment. The normal rodent chow was removed from the mouse cages during access to the diet. The diet was provided in drinking bottles fitted with ball-point sipper tubes to reduce spillage. The mice were habituated to the normal diet for the first 3 days and were then given access to the alcohol diet or normal diet for the next 2 weeks. On the day of bilateral ischemia–reperfusion (IR) surgery, the diet was removed, and the mice were continuously fed laboratory chow for the rest of the experiments. Blood was taken from the tail vein of each mouse to measure the blood alcohol concentration. Approximately 20 μl of whole blood was collected into specialized capillary tubes, and the blood alcohol concentration was determined using the Analox alcohol analyzer (Analox Instruments, Lunenburg, MA, USA) at the end of the treatment. The average blood alcohol concentration was approximately 98±3.6 mg dl^−1^ at the end of the treatment.

### Bilateral IR surgery

Bilateral IR was performed based on an established protocol as described previously^43^ on male *Arrb2*^−/−^ and WT C57BL/6J mice. Briefly, both renal pedicles were exposed by flank incisions and clamped using micro aneurysm clamps for 15 min under pentobarbital anesthesia (60 mg kg^−1^, i.p.). At the end of the ischemic period, the clamps were released for reperfusion. Sham groups of mice underwent surgery to expose the renal pedicles without clamping. To examine the role of GSK3β in the effects of chronic alcohol exposure on acute kidney injury, IR mice received a vehicle injection (1% dimethylsulphoxide; 1 ml kg^−1^) or SB216763 (20 mg kg^−1^; a potent inhibitor of GSK-3, Tocris Bioscience, Shanghai, China) 30 min before clamping.

### Measurement of creatinine and blood urea nitrogen

Blood was collected from the tail vein, and the plasma was used to measure BUN and plasma creatinine using a QuantiChrom Urea Assay Kit from BioAssay Systems (Hayward, CA, USA) and a Creatinine Assay Kit (Abcam, Shanghai, China) respectively, following the manufacturer’s instructions.

### Quantitative PCR

Total RNA was extracted by Trizol (Invitrogen, Pleasanton, CA, USA) and reverse transcribed into complementary DNA using kits (Applied Biosystems, Waltham, MA, USA). The PCR with reverse transcription primers for GAPDH, collagen 1a1, TGFβ1, CCN2 and CCN3 are listed in Table 1. The PCR analyses were performed using a SYBR Green-based system (Applied Biosystems), and the expression levels were normalized to GAPDH and expressed as relative levels to sham controls. Each gene was analyzed in triplicate. Calculations were performed using the comparative-CT method.

### Western blotting

Kidney tissues were lysed in RIPA buffer and loaded onto SDS-polyacrylamide gel electrophoresis gels, transferred to nitrocellulose membranes and blocked with 5% milk in TBST. The membranes were probed with primary antibodies against Akt, pAkt (Thr308), GSK3β, pGSK3β (Ser9), β-arrestin 2 (Cell Signaling Technology, Shanghai, China), collagen I, TGFβ1, CCN2, CCN3 (Abcam) and β-actin (Abcam), followed by washing with TBST and incubation with a horseradish peroxidase-conjugated secondary antibody (Sigma-Aldrich, Shanghai, China).

### Histology

Renal tissues were fixed in 4% paraformaldehyde in 1 × phosphate-buffered saline (pH 7.4) for 24 h at 4 °C. After fixation, the tissue samples were routinely processed and embedded in paraffin wax (TissuePrep II, Fisher Scientific, Shanghai, China). Tissue sections of 5-μm thickness were cut, placed on SuperfrostPlus slides (Fisher Scientific), de-paraffinized and rehydrated through a descending alcohol series. The tissue sections were routinely stained with Mayer’s hematoxylin and Putt’s eosin (H&E). Masson’s trichrome staining was also performed using a staining kit (Abcam) to visualize fibrotic tissue.

### Immunohistochemistry

Paraffin sections were de-paraffinized, washed in phosphate-buffered saline containing 0.1% Tween 20 and then blocked in 10% normal goat serum. Primary antibodies against collagen I, α-smooth muscle actin (SMA) and fibronectin were applied to the sections and incubated at 4 °C overnight. The sections were then washed in Tween 20-buffered saline and incubated with a biotinylated secondary antibody. After further washes with Tween 20-buffered saline, the sections were incubated with an avidin-biotinylated horseradish peroxidase complex. Finally, the sections were rewashed and developed by diaminobenzidine tetrahydrochloride (Sigma, Shanghai, China).

### Statistical analysis

The data are expressed as the mean±s.d. All data analyses were performed using SPSS 20.0 statistical software. Multiple-group comparisons were analyzed by an analysis of variance (ANOVA) with a *post hoc* Tukey’s test, and *P*<0.05 was considered statistically significant.

## Results

### Chronic alcohol exposure potentiated bilateral IR-induced renal fibrosis and renal function impairment

**Figure 2:**
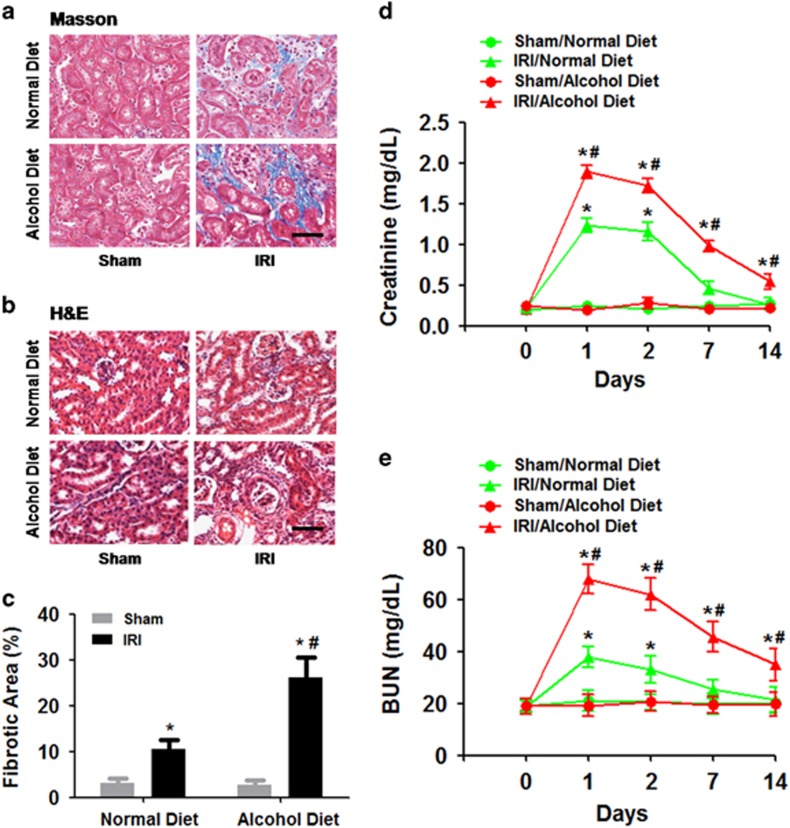
Effects of chronic alcohol exposure on bilateral ischemia–reperfusion (IR)-induced renal fibrosis and renal function impairment. (**a**) Representative images of Masson’s trichrome staining. (**b**) Representative images of H&E staining. (**c**) Fibrosis scores were assigned based on Masson’s trichrome staining. (**d**) Plasma creatinine levels. (**e**) Blood urea nitrogen (BUN) levels. *P<0.05 compared to the sham group, ^#^*P*<0.05 compared to the normal diet group; *n*=8 mice per group. Scale bar represents 50 μm. All tissue samples were collected at day 14 after IR.

We first examined the impact of chronic alcohol exposure on the consequences of bilateral IR-induced AKI. We found that 2 weeks of a 4.5% alcohol diet treatment did not induce renal fibrosis in sham mice compared with a normal diet treatment (Figures 2a–c). However, mice with IR-induced AKI developed renal fibrosis 14 days later compared with the sham controls (ANOVA, surgery main and interaction effects, F_(1,28)_=28.77–34.19, *P*<0.0001; Tukey’s test, *P*<0.05; Figures 2a–c). Furthermore, the chronic alcohol treatment enhanced IR-induced renal fibrosis in mice 14 days after surgery compared with the normal diet treatment (Tukey’s test, *P*<0.05; Figures 2a–c). Dilated tubules and tubular atrophy were observed in the IR groups using H&E staining, and the alcohol diet treatment group exhibited enhanced injury compared with the normal diet treatment group (Figure 2b).

Blood creatinine levels significantly increased in WT mice as early as 24 h after IR (ANOVA, surgery main and interaction effects, F_(1–4,28–108)_=26.54–38.92, *P*<0.0001; Tukey’s test, *P*<0.05; Figure 2d). However, the increased creatinine levels were exacerbated in mice with chronic alcohol exposure compared with the normal diet treatment group (ANOVA, treatment main effect, F_(1,28)_=30.27, *P*<0.0001; Tukey’s test, *P*<0.05; Figure 2d). Furthermore, creatinine levels declined over 14 days after IR. Mice given the normal diet exhibited normal creatinine levels 14 days after IR (Figure 2d). However, the creatinine levels in mice with chronic alcohol exposure remained elevated 14 days after IR compared with the normal diet treatment group (Tukey’s test, *P*<0.05; Figure 2d).

Similarly, BUN levels significantly increased in WT mice as early as 24 h after IR (ANOVA, surgery main and interaction effects, F_(1–4,28–108)_=29.41–40.03, *P*<0.0001; Tukey’s test, *P*<0.05; Figure 2d). However, BUN levels were exacerbated in mice with chronic alcohol exposure compared with the normal diet treatment group (ANOVA, treatment main effect, F_(1,28)_=33.18, *P*<0.0001; Tukey’s test, *P*<0.05; Figure 2d). Furthermore, BUN levels declined over 14 days after IR. Mice given the normal diet treatment exhibited normal BUN levels 14 days after IR (Figure 2d). However, the BUN levels in mice with chronic alcohol exposure were still enhanced 14 days after IR compared with the normal diet treatment group (Tukey’s test, *P*<0.05; Figure 2e).

### Chronic alcohol exposure increased bilateral IR-induced extracellular matrix deposition and an increased myofibroblast population

**Figure 3:**
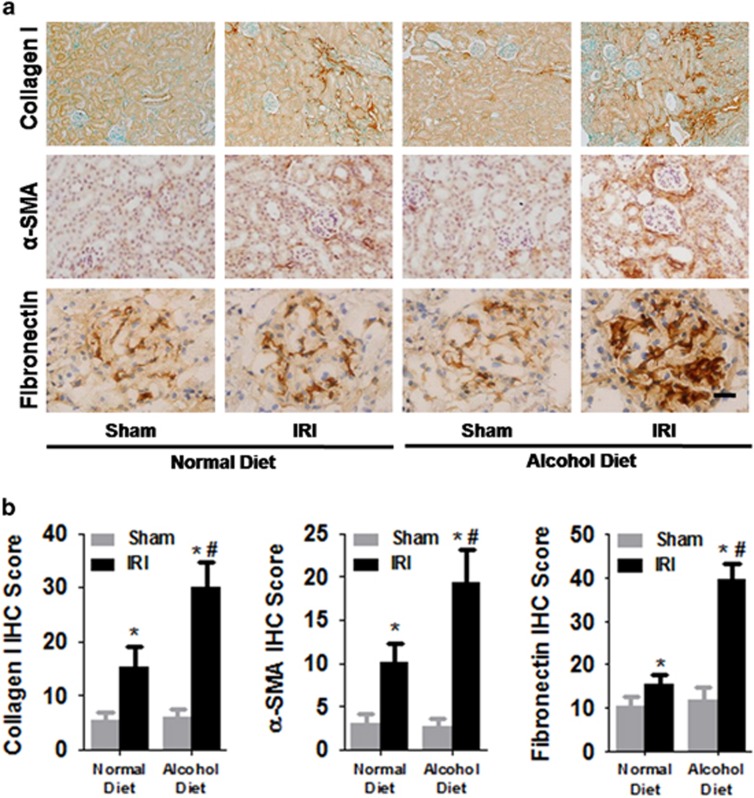
Effects of chronic alcohol exposure on bilateral ischemia–reperfusion (IR)-induced extracellular matrix deposition and myofibroblast population. (**a**) Representative images of immunostaining for fibronectin, collagen-1 and α-SMA in mouse renal tissues. (**b**) The area % stain represents the ratio of the summed absolute areas of staining versus the total tissue. *P<0.05 compared to the sham group, ^#^*P*<0.05 compared to the normal diet group; *n*=8 mice per group. Scale bar represents 50 μm. All tissue samples were collected at day 14 after IR.

Renal fibrosis is characterized by excessive extracellular matrix (ECM) remodeling. To determine the effect of chronic alcohol treatment on ECM deposition and the myofibroblast population, we examined the expression levels of collagen-1 and fibronectin, which are two major matrix components. We also determined the myofibroblast population in the kidneys by measuring α-SMA expression levels, because active myofibroblasts are a major source of collagen and fibronectin. We found that 2 weeks of the 4.5% alcohol diet treatment did not induce excessive ECM deposition in sham mice compared with the normal diet treatment (Figures 3a and b). However, mice with IR-induced AKI exhibited enhanced collagen-1 and fibronectin deposition as well as α-SMA expression at 14 days post IR compared with sham controls (ANOVA, surgery main and interaction effects, F_(1,28)_=26.47–31.02, *P*<0.0001; Tukey’s test, *P*<0.05; Figures 3a and b). Furthermore, the chronic alcohol treatment enhanced IR-induced collagen-1 and fibronectin deposition as well as α-SMA expression at 14 days post IR 14 compared with the normal diet treatment (Tukey’s test, *P*<0.05; Figures 3a and b).

### Chronic alcohol exposure increased bilateral IR-induced expression of fibrosis-related genes in the kidneys

**Figure 4:**
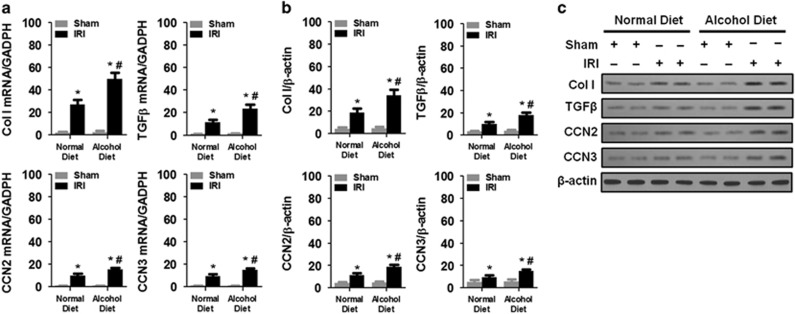
Chronic alcohol exposure increased the bilateral IR-induced expression of fibrosis-related genes in the kidney. (**a**) mRNA expression of Col I, TGFβ, CCN2 and CCN3. The data were normalized to GAPDH. (**b**) Protein levels of Col I, TGFβ, CCN2 and CCN3. (**c**) Representative western blots. *P<0.05 compared to the sham group, ^#^*P*<0.05 compared to the normal diet group; *n*=8 mice per group. All tissue samples were collected at day 14 after IR.

To further examine the effects of chronic alcohol exposure on renal fibrosis, we determined the mRNA and protein expression levels of several fibrosis-related genes, including *Col I*, *TGFβ*, *CCN2* and *CCN3.* We found that 2 weeks of the 4.5% alcohol diet treatment did not induce the mRNA or protein expression of *Col I*, *TGFβ*, *CCN2* and *CCN3* compared with the normal diet treatment (Figures 4a–c). However, mice with IR-induced AKI exhibited enhanced mRNA and protein expression of *Col I*, *TGFβ*, *CCN2* and *CCN3* at 14 days post IR compared with the sham controls (ANOVA, surgery main and interaction effects, F_(1,28)_=27.44–36.01, *P*<0.0001; Tukey’s test, *P*<0.05; Figures 4a–c). Furthermore, the chronic alcohol treatment promoted IR-induced mRNA or protein expression of *Col I*, *TGFβ*, *CCN2* and *CCN3* at 14 days post IR compared with the normal diet treatment (Tukey’s test, *P*<0.05; Figures 4a–c).

### Chronic alcohol exposure enhanced the expression of β-arrestin 2 and the activation of Akt and GSK3β

**Figure 5:**
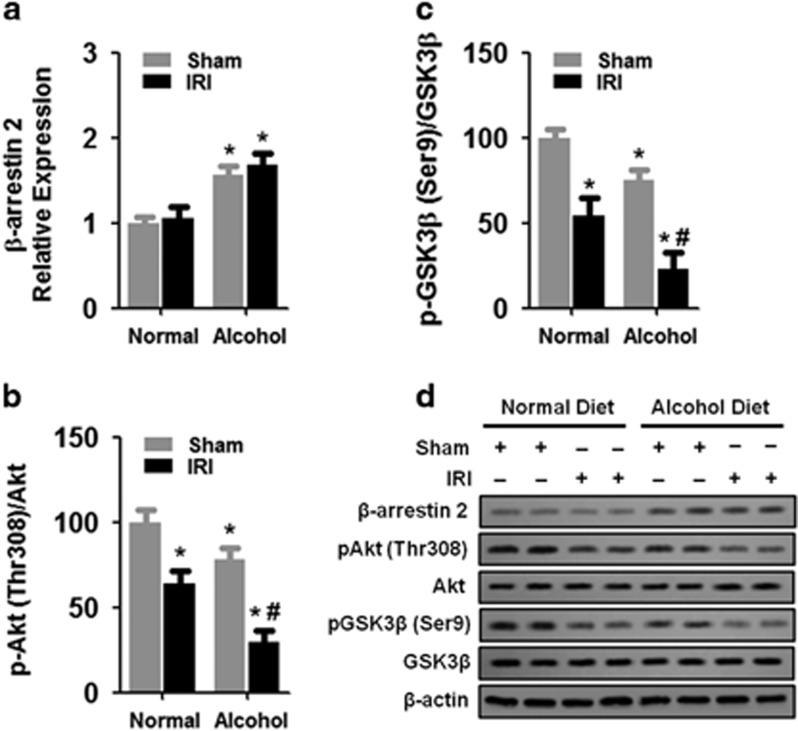
Effects of chronic alcohol exposure on β-arrestin 2 expression and Akt and GSK3β activation. (**a**) Protein levels of β-arrestin 2. The data were normalized to β-actin and then to the sham/normal diet group. (**b**) Protein levels of Akt and p-Akt (Thr308). The p-Akt/Akt ratio was normalized to that of the sham/normal diet group. (**c**) Protein levels of GSK3β and p-GSK3β (Ser9). The p-GSK3β/GSK3β ratio was normalized to that of the sham/normal diet group. (**d**) Representative western blot. *P<0.05 compared to the sham/normal diet group, ^#^*P*<0.05 compared to the sham/alcohol diet group; *n*=8 mice per group. All tissue samples were collected at day 14 after IR.

Given that β-arrestin 2 is critical in the regulation of GSK3 activation, which is involved in IR-induced renal fibrosis, we determined the effect of chronic alcohol treatment on the protein levels of β-arrestin 2, Akt, pAkt (Thr308), GSK3β and pGSK3β (Ser 9). We found that 2 weeks of the 4.5% alcohol diet treatment enhanced the protein expression of β-arrestin 2 in the kidneys compared with the normal diet treatment (Figures 5a and d). However, IR-induced AKI did not alter the protein expression of β-arrestin 2 at 14 days post IR compared with the sham controls (ANOVA, surgery main and interaction effects, F_(1,28)_=29.11–33.19, *P*<0.0001; Tukey’s test, *P*<0.05; Figures 5a and d).

In addition, chronic alcohol exposure did not alter the protein levels of Akt in the kidneys compared to the normal diet treatment (Figures 5b and d). Moreover, IR-induced AKI did not alter the protein levels of Akt in the kidneys compared with sham controls (Figures 5b and d). However, 2 weeks of the 4.5% alcohol diet treatment significantly decreased the protein levels of pAkt in the kidneys compared with the normal diet treatment (ANOVA, treatment main and interaction effects, F_(1,28)_=36.47–46.33, *P*<0.0001; Tukey’s test, *P*<0.05; Figures 5b and d). Furthermore, IR-induced AKI reduced the protein levels of pAkt in the kidneys compared with sham controls (Tukey’s test, *P*<0.05; Figures 5b and d). Notably, IR-induced AKI reduced the protein levels of pAkt in the kidneys to a greater extent compared with the normal diet treatment (Tukey’s test, *P*<0.05; Figures 5b and d).

Similarly, 2 weeks of the 4.5% alcohol diet treatment did not alter the protein levels of GSK3β in the kidneys compared with the normal diet treatment (Figures 5c and d). In addition, IR-induced AKI did not alter the protein levels of GSK3β in the kidneys compared with sham controls (Figures 5c and d). However, 2 weeks of the 4.5% alcohol diet treatment significantly decreased the protein levels of pGSK3β in the kidneys compared with the normal diet treatment (ANOVA, treatment main and interaction effects, F_(1,28)_=30.04–38.93, *P*<0.0001; Tukey’s test, *P*<0.05; Figures 5c and d). Furthermore, IR-induced AKI reduced the protein levels of pGSK3β in the kidneys compared with sham controls (Tukey’s test, *P*<0.05; Figures 5c and d). Similar to the pAkt results, IR-induced AKI reduced the protein levels of pGSK3β in the kidneys to a greater extent compared with the normal diet treatment (Tukey’s test, *P*<0.05; Figures 5c and d).

### GSK3 inhibition alleviated bilateral IR-induced renal fibrosis and renal function impairment

**Figure 6:**
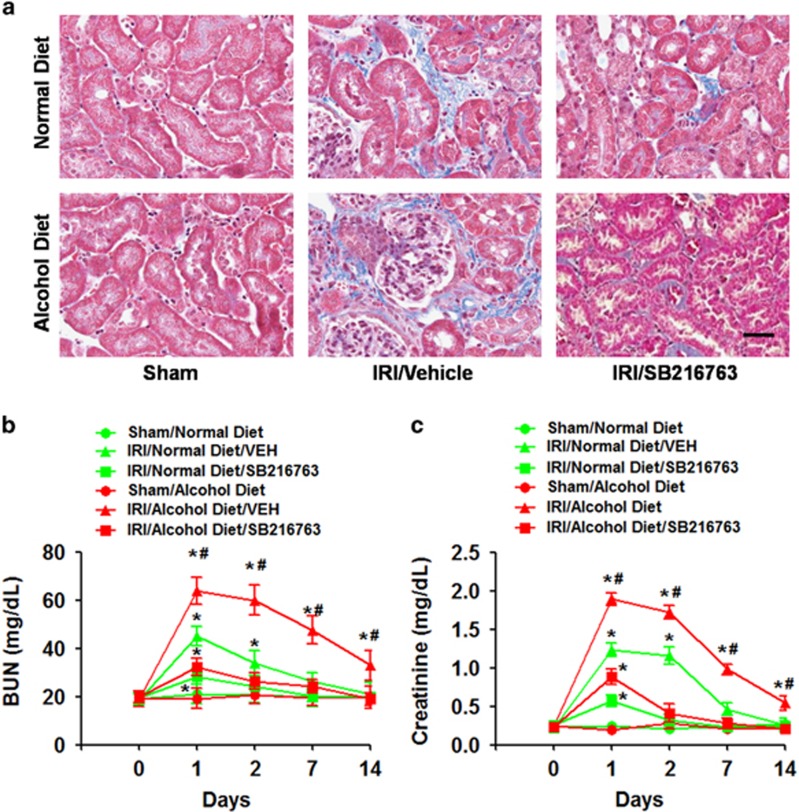
Effects of SB216763 on bilateral IR-induced renal fibrosis and renal function impairment. (**a**) Representative images of Masson’s trichrome staining. (**b**) Blood urea nitrogen (BUN) levels. (**c**) Plasma creatinine levels. *P<0.05 compared to the sham group, ^#^*P*<0.05 compared to the normal diet group; *n*=8 mice per group. Scale bar represents 50 μm. All tissue samples were collected at day 14 after IR.

To determine whether GSK3 plays a critical role in mediating the effects of chronic alcohol treatment on IR-induced renal injury, we treated WT mice with SB216763 (20 mg kg^−1^, IP), a GSK inhibitor, 30 min before IR and examined its effects on subsequent IR-induced renal fibrosis and renal function impairment. Replicating our previous findings, we found that the chronic alcohol treatment enhanced IR-induced renal fibrosis in mice at 14 days post IR compared with the normal diet treatment (Figure 6a). However, mice pretreated with SB216763 (20 mg kg^−1^, IP) exhibited much less renal fibrosis compared with vehicle-pretreated mice (ANOVA, treatment main and interaction effects, F_(1,28)_=19.47–25.38, *P*<0.001; Tukey’s test, *P*<0.05; Figure 6a).

Similarly, we found that the chronic alcohol treatment enhanced the IR-induced elevation of BUN and creatinine levels in mice compared with the normal diet treatment (Figures 6b and c). However, mice pretreated with SB216763 (20 mg kg^−1^, i.p.) exhibited much less enhancement of BUN and creatinine levels compared with vehicle-pretreated mice (ANOVA, treatment main and interaction effects, F_(1,28)_=17.21–20.87, *P*<0.01; Tukey’s test, *P*<0.05; Figures 6b and c).

### *Arrb2*^−/−^ mice exhibited resistance to IR-induced renal fibrosis and renal function impairment following chronic alcohol exposure

**Figure 7:**
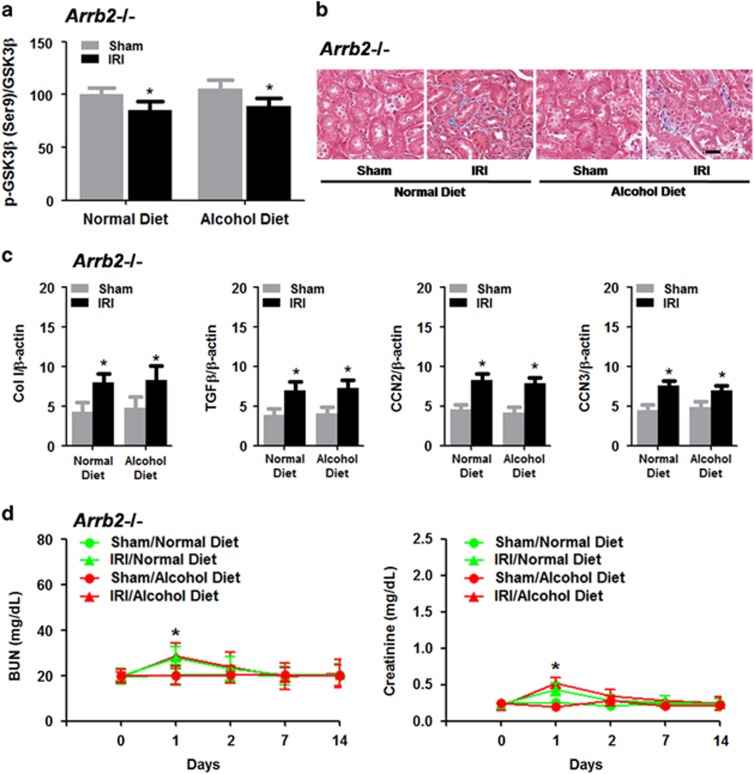
IR-induced renal fibrosis and renal function impairment following chronic alcohol exposure in *Arrb2*^−/−^ mice. (**a**) Effects of IR-induced GSK3β activation in *Arrb2*^−/−^ mice after chronic alcohol exposure. (**b**) Representative images of Masson’s trichrome staining in *Arrb2*^−/−^ mice after chronic alcohol exposure. (**c**) Protein levels of Col I, TGFβ, CCN2 and CCN3. (**d**) Blood urea nitrogen (BUN) and plasma creatinine levels. *P<0.05 compared to the sham group; *n*=8 mice per group. Scale bar represents 50 μm. All tissue samples were collected at day 14 after IR.

Given that we observed enhanced β-arrestin 2 levels in the kidneys after chronic alcohol exposure, we decided to determine whether β-arrestin 2 plays a critical role in mediating the effects of chronic alcohol treatment on IR-induced renal injury. We first found that sham-operated *Arrb2*^−/−^ mice exhibited resistance to chronic alcohol treatment-induced GSK3β activation (that is, reduced pGSK3β (Ser9)/GSK3β ratio; Figure 7a), although IR induced a mild increase in GSK3β activation in chronic alcohol-treated *Arrb2*^−/−^ mice, similar to that observed in normal diet-treated *Arrb2*^−/−^ mice (Figure 7a). Similarly, the chronic alcohol treatment did not exaggerate renal fibrosis at 14 days post-IR in *Arrb2*^−/−^ mice (Figure 7b). Furthermore, we confirmed that the chronic alcohol treatment did not alter the IR-induced protein expression of *Col I*, *TGFβ*, *CCN2* and *CCN3* at 14 days post IR compared with the normal diet treatment (Figure 7c), although IR induced a mild increase in the protein expression of *Col I*, *TGFβ*, *CCN2* and *CCN3* in chronic alcohol-treated *Arrb2*^−/−^ mice, similar to that observed in normal diet-treated *Arrb2*^−/−^ mice (Figure 7c). Finally, we showed that *Arrb2*^−/−^ mice exhibited resistance to IR-induced increases in BUN and creatinine levels, and the chronic alcohol treatment did not alter the IR-induced increases in BUN and creatinine levels compared with normal diet-treated *Arrb2*^−/−^ mice (Figure 7d).

## Discussion

In this study, we examined the effects of chronic alcohol intake on the development of renal fibrosis following AKI in an animal model of bilateral renal IRI. We first found that chronic alcohol exposure exacerbated bilateral IR-induced renal fibrosis and renal function impairment. This phenomenon was associated with increased bilateral IR-induced extracellular matrix deposition and an increased myofibroblast population as well as increased bilateral IR-induced expression of fibrosis-related genes in the kidneys. To explore the mechanisms underlying this phenomenon, we showed that chronic alcohol exposure enhanced β-arrestin 2 expression and Akt and GSK3β activation in the kidneys. Importantly, pharmacological GSK3 inhibition alleviated bilateral IR-induced renal fibrosis and renal function impairment. Furthermore, we demonstrated that *Arrb2*^−/−^ mice exhibited resistance to IR-induced renal fibrosis and renal function impairment following chronic alcohol exposure, and these effects were associated with attenuated GSK3β activation in the kidneys. Taken together, our results suggest that chronic alcohol exposure may exacerbate AKI via β-arrestin 2/Akt/GSK3β-mediated signaling in the kidney. Numerous studies have demonstrated that acute alcohol pretreatment improves IR-induced injury and alleviates the subsequent dysfunction of multiple organs, including the heart, brain, liver, intestine and kidney.^44, 45, 46, 47, 48, 49^ In fact, ethanol preconditioning experiments in animal models have shown that ethanol prevents post-ischemic adhesive interactions between leukocytes and endothelial cells, which can result in organ dysfunction and death.^50, 51^ Furthermore, the anti-adhesive and anti-inflammatory effects of acute alcohol exposure are dependent on aldehyde dehydrogenase, which eliminates the toxic aldehydes produced by IR to prevent lipid peroxidation.^48, 50, 52^ However, studies have shown that acute alcohol exposure may have different effects than chronic alcohol exposure. For example, acute alcohol exposure has anti-inflammatory effects on lipopolysaccharide-induced inflammation in human monocytic cells *in vitro* and *in vivo*.^53, 54, 55^ In contrast, chronic alcohol exposure enhances inflammatory cytokine gene expression in humans and rodent models.^56, 57, 58^ Similarly, our results showed that chronic alcohol exposure may exacerbate AKI via activation of GSK3β-mediated signaling in the kidney, which is likely mediated by the β-arrestin 2/Akt signaling pathway. Consistently, previous studies have shown that chronic ethanol intake can decrease the phosphorylation of Akt at Thr308 and increase phosphorylation at Ser473, which results in decreased GSK3β phosphorylation in rat liver tissues.^31^ In addition, ethanol can induce the dephosphorylation of GSK3β at Ser9 in the cerebral cortex of mice but has little effect on the expression of total GSK3β.^32^ Thus, our study adds to the literature by demonstrating for the first time that chronic alcohol exposure exacerbates IR-induced injury in the kidney.

It should be noted that withdrawal from chronic alcohol intake may also contribute to the development of renal fibrosis after AKI. In fact, studies have shown that the activity of the autonomic nervous system shifts to favor sympathetic activity during alcohol withdrawal.^59, 60^ As a result, laboratory animals undergoing withdrawal experience changes in cardiovascular functions, such as elevated heart rate and blood pressure. It has been shown that renal sympathetic nerve activity is significantly augmented during renal ischemia.^61, 62^ Renal denervation or administration of pentolinium, a ganglion blocking agent, before ischemia attenuates the ischemia/reperfusion-induced renal dysfunction and histological damage.^61, 63^ Thus, it is likely that alcohol withdrawal-induced activation of renal sympathetic nerve activity also contributes to the subsequent deterioration of renal functions.

Consistent with previous studies showing that GSK3β is expressed in myofibroblasts in mouse kidneys,^30^ the present study also showed that GSK3 inhibition reduced the expression of α-SMA, a marker of myofibroblast population, and inhibited the expression of several fibrosis-related genes, including TGFβ, connective tissue growth factor (CCN2), and CCN3. As a critical mediator of fibrosis, TGFβ is well known to promote fibroblast activation, proliferation, migration and ECM synthesis.^64, 65^ However, the role of GSK3 in regulating TGFβ signaling seems to be dependent on tissues or cells. Specifically, pharmacological GSK3 inhibition attenuates TGFβ1-mediated signaling and ECM accumulation in cultured renal glomerular mesenchymal cells and in lung, gingival, corneal and skin fibroblasts.^66, 67, 68, 69, 70^ In contrast, other studies have shown that GSK3 inhibition increases TGFβ1-induced β-catenin and Snail accumulation in a unilateral ureteral obstruction model and renal epithelial cells *in vitro*.^71, 72, 73, 74^ Nonetheless, our results are consistent with a previous study showing that GSK3 inhibition significantly reduced the expression of TGFβ1 in a rodent IR model of AKI.^30^ In addition, the increased CCN2 and CCN3 expression in chronic alcohol-treated mice after renal IR may be due to enhanced GSK3 activation. We showed that inhibition of GSK3 reduced TGF-β, CCN2 and CCN3 expression. While we did not examine the role of TGFβ in the regulation of CCN2 and CCN3 expression in chronic alcohol-treated mice after renal IR, previous studies have shown that TGFβ1 strongly induces the expression of CCN2.^75, 76^ Furthermore, GSK-3β activity is required for TGFβ_1_-mediated CCN2 expression in human gingival fibroblasts.^66^ Future studies will be important to examine the role of TGFβ in IR-induced injury in the kidney following chronic alcohol exposure.

One important finding in the present study is that β-arrestin 2 plays a critical role in the regulation of IR-induced injury in the kidney following chronic alcohol exposure. Increasing evidence reveals that β-arrestin 2 can modulate the activation of the serine/threonine kinase Akt.^77, 78^ Activated Akt phosphorylates downstream GSK3β at serine 9, resulting in GSK3β inactivation.^79, 80^ Our study showed that neither chronic alcohol exposure nor renal IR-induced AKI altered the expression of GSK3β, but both reduced GSK3β phosphorylation at serine 9, indicating enhanced GSK3β activation. Similarly, previous studies have shown that the presence of β-arrestin 2 reduces Akt phosphorylation, which is associated with increased liver injury.^81^ While the mechanisms involved in the increased GSK3β activation after chronic alcohol exposure remain unclear, our study suggests that chronic alcohol exposure may enhance GSK3β activation by increasing β-arrestin 2 expression. Hence, additional studies will be needed to examine the specific role of Akt in mediating the regulation of GSK3β activation via β-arrestin 2.

In conclusion, our study revealed that chronic alcohol exposure may potentiate AKI via β-arrestin 2/GSK3β-mediated signaling in the kidney. However, the pathogenesis of acute renal injury involves complex multi-cellular interactions within the heterogeneous renal tissue. Hence, future studies will be necessary to examine what might exacerbate acute kidney injury, which may allow the development of more specific and effective pharmacotherapies for the prevention of AKI.

## Figures and Tables

**Table 1:** Primers used for RT-PCR

*Gene name*	*Primers*
Collagen-1a1	F AGACATGTTCAGCTTTGTGGAC R GCAGCTGACTTCAGGGATG
TGF-β1	F TGAGTGGCTGTCTTTTGACG R AGCCCTGTATTCCGTCTCCT
CCN2	F CTCGGACTGTGATGCCTTAAT R TGGATCCACACCTTGCATTTA
CCN3	F GAAGATTCCACGCCAATTCATC R GATCTGCCGGTTTCTCTTAGTC
GAPDH	F TGCACCACCAACTGCTTAGC R GGCATGGACTGTGGTCATGAG

Abbreviation: RT-PCR, PCR with reverse transcription.
